# 

**Published:** 1892-04-23

**Authors:** 


**Notices of Births, Deaths, and Marriages,** are inserted in this column at a charge of 2s. 6d. for an announcement not exceeding four lines.

### NOTICE TO CORRESPONDENTS.

All MS., Letters, Books for Review, and other matters intended for the Editor should be addressed The Editor, The Lodge, Porchester Square, London, W.

The Editor cannot undertake to return rejected M.S., even when accompanied by stamped directed envelope.

**Notice to Advertisers and Subscribers.**

All Advertisements, Orders for Copies of The Hospital, and Business Communications should be addressed to The Publisher, *not to the Editor*, The Hospital, 140, Strand, W.C.

The Cost of Subscription, post free, is as follows:—Per week, 2½d.; per quarter, 2s. 6d.; per half-year, 5s.; per annum, 8s. 6d. Foreign:—Per annum, 10s. 8d.; payable, in all cases, in advance.

**"Burdett's Hospital Annual," 1891—1892.**

Third year of publication. 600 pp. Boards, gilt lettering. A most complete guide to British and Colonial Hospitals, Dispensaries, Nursing and Convalescent Institutions, and Asylums. Price 3s. 6d. Published by The Scientific Press (Limited), 140, Strand, W.C.

**NOTICE.—The Index for Vol. X. (ending September, 1891), is on sale at the Office of this Journal, price 2½d., post free.**

### SCRAPS AND GLEANINGS.

THE Queen has contributed £25 to the Royal Medical Benevolent College, Epsom.

THE Skinners' Company have sent a donation of £21 to the Irish Distressed Ladies' Fund.

FIVE persons were severely injured at Leicester on Saturday by the overturning of an omnibus.

THE epidemic of diphtheria is decreasing at Heywood, and the public schools are being reopened.

SIR ANDREW CLARK has been elected, for the fifth time, President of the Royal College of Physicians of London.

No fewer than 5,424 emigrants from Europe arrived last Thursday at New York, the largest number ever recorded.

THE Worshipful Company of Clothworkers have given a donation of £50 to the new Hospital for Women, 144, Euston Road.

THE late Dr. H. Elthington Price, of No. 1, Tisbury-road, West Brighton, has left a legacy of £105, free of duty, to the Hospital Sunday Fund.

AN epidemic of scarlatina prevails in Faversham, and the Medical Officer of Health has advised the closing of the public elementary schools.

THE Clothworkers' Company have given £25, and the Mercers' Company 20 guineas to the funds of the National Industrial Home for Crippled Boys, Kensington.

MRS. DARBYSHIRE and Mrs. Davies were among the successful candidates elected as Guardians for Hove in the S eyning Union. This is the first time that ladies have been elected to this Board.

THE death is announced of Surgeon-General Edward Courtenay Thorp, who died at Folkestone, in his 71st year. He served at the Siege of Mooltan, the Battle of Goozerat, and in the Bhootan Campaign.

BRIGADE SURGEON LIEUTENANT-COLONEL C. E. HARRISON, of the Brigade of Foot Guards, has been selected to act as medical representative of the British Army at the Congress on Surgery to be held at Paris, commencing on the 18th inst.

THE Pharmaceutical Society obtained judgment for £32 in the Penzance County Court against Richard Redland, trading as a chemist in Penzance, being penalties under the Pharmacy Act for selling and dispensing poisons, and styling himself a chemist.

THE Sanitary Committee of the Leeds Corporation have purchased for £10,000, the Manston Hall Estate, near Leeds, for the purposes of a permanent hospital for infectious diseases. The house is beautifully situated, and the property consists also of 97 acres of grass land.

THE Duke of Westminster has presented the Edgar Field, at Handbridge, Chester, for the recreation of the poor children of that suburb, and has, it is understood, further intimated his intention of placing £1,000 to the credit of the Corporation of Chester as an endowment fund for keeping the grounds in order.

SIR WILLIAM BOWMAN, F.R.S., who died recently, in 1842 gained the Royal Medal for Physiology, and three years later, being then only twenty-nine, was Professor of Physiology and Morbid Anatomy at King's College and Vice-President of the Royal Society. In 1884 he was elected a Fellow of the Royal College of Surgeons.

DURING the nine months last year, March 26th to November 13th, that the Ilkley Hospital and Convalescent Home received patients, the number of admissions, including renewals, was 1,120. A pleasing feature in the year's receipts was the large increase in church collections, namely £74 18s. 6d., as compared with £29 6s. 9d. in 1890.

AN inquest was held at Liverpool, touching the death of Martha Roberts. Deceased cut her hand a few weeks ago, and to stop the bleeding, adopted the housewife's plan of using a cobweb. Blood poisoning set in—presumably because there was dirt on the cobweb—and the woman died. The jury returned a verdict in accordance with the evidence.

IT has been decided to enlarge the Royal Ear Hospital in Soho Square, so as to adapt it to the growing requirements of these days. The hospital was founded in 1816, when diseases of the ear were much less understood than they are now, and at that time King George, who himself suffered from deafness and earache, took a special interest in its formation.

MR. CHARLES HENRY WAGNER, merchant, of Birmingham, has left the following bequests to Birmingham charities: Midland Institute, £500; General Hospital, £500; Queen's Hospital, £500; General Dispensary, £300; the Children's Hospital, the Institution for the Blind, and the Deaf and Dumb Institution, £100 each; and the German Hospital, Dalston, £50.

AT a special meeting of the Metropolitan Asylums Board held recently, a large deputation of the inhabitants of Stoke Newington waited upon the Board to urge them not to purchase the proposed site for a fever hospital near Clissold Park. The site occupies nine acres, and the price is £36,200, or £10,000 more than it was bought for last November. After much discussion, it was finally decided not to buy the land.

THE financial year of the Meath Hospital and County Dublin Infirmary, opened with a balance against the hospital of £523 18s. 5d.; and the expenditure for the year amounted to £4,837 14s. 11d., making a total of £5,361 13s. 4d. The income from all sources amounted to £5,184 15s. 7d. At the close of the year, therefore, the debit balance was reduced to £176 17s. 9d. Total number of patients admitted during the year was 1,363.

MISS LOUISA HANNAH FAWSETT BENNETT, formerly of The Grange, Cheshire, bequeaths £6,000 to the Manchester Royal Infirmary; £5,000 to the British Home for Incurables (Clapham Rise), on condition that a ward is maintained bearing the name of "Hannah Fawsett Bennett," in memory of her late mother; £3,000 to the Hospital for Consumption and Diseases of the Chest, Brompton; £2,000 each to the Manchester Blind Asylum (Old Trafford), the Establishment for Gentlemen (90, Harley Street), and the Cancer Hospital (Brompton); £1,000 each to the Royal Association in aid of the Deaf and Dumb (27[illegible], Oxford Street), the Middlesex Hospital, St. Thomas's Hospital, the Clergy Ladies' Home (Westbourne Park).

### THE STUDENT OF MEDICINE.

[All communications for this Supplement should be addressed to The Editor of The Hospital, at 140, Strand, with the words, "Students' Column," written in the left hand top corner of the envelope.]

### APPOINTMENTS.

Bodmer, R., F.C.S., F.I.C., reappointed Public Analyst for the Bermondsey Local Board. Clarkson, R. D., M.B., C.M. Edin., appointed Resident Physician to the Royal Edinburgh Hospital for Sick Children. Davies, Edward, M.D.St.And., M.R.C.S.Eng., reappointed Medical Officer to the Wrexham Fever Hospital. Dowden, J. W., M.B., C.M.Edin., appointed Resident Physician at the Royal Edinburgh Hospital for Sick Children. Frankish, Thomas, M.B., C.M.Edin., appointed Dispensary Surgeon to the Bradford Infirmary, vice H. N. D. Milligan, M.B., C.M.Edin. Harris, Thomas, M.D.Lond., M.R.C.P., M.R.C.S., appointed Honorary Physician to the Manchester Royal Infirmary, vice James Ross, LL.D., M.D.Aberd. Horrocks, W., F.R.C.S., appointed Honorary Surgeon to the Bradford Infirmary, vice Dr. Rabagliati. Lees, C. F., M.B., C.M.Edin., appointed Assistant Medical Officer at the Hope Hospital, Manchester. Lumley, Bartholomew, M.R.C.S.Eng., L.S.A., reappointed Medical Officer of Health to the Northallerton Local Board. Morris, W. J., L.R.C.P.Edin., M.R.C.S., appointed Medical Officer for the Tremadoc Sanitary District of the Festiniog. Munden, Charles, M.R.C.S.Eng., L.S.A., reappointed Medical Officer of the No. 3 Ilminster District of the Chard Union. Opie, Edward A., M.B.Durh., M.R.C.S.Eng., appointed Medical Officer for the No. 2B District of the East Preston Union. Pratt, John Wyatt, L.R.C.P.Edin., M.R.C.S. Eng., appointed Medical Officer of Health to the Wiveliscombe Local Board. Richardson, W. J., M.R.C.S., L.R.C.P.Lond., appointed Medical Officer to the Brentford Post Office. Roberts, Edward C., M.R.C.S., reappointed Medical Officer of Health for Southgate. Robinson, Frank, M.B., Ch.B., appointed Assistant Medical Officer to the Workhouse Infirmary, Crumpsall, and Resident Assistant Medical Officer to the Casual and Receiving Wards, New Bridge Street Manchester. Sinclair, Walter W., M.B., C.M.Aberd., appointed Junior House-Surgeon to the Birmingham and Midland Eye Hospital, Birmingham. Wade, Newton, L.R.C.P.Lond., M.R.C.S.Eng., reappointed Medical Officer for the No. 2 District of the Shepton Mallet Union. Webber, W. W., L.R.C.P.Edin., M.R.C.S.Eng., reappointed Medical Officer of the Crewkerne District of the Chard Union.

### VACANCIES.

### Resident Medical Officers.

The following vacancies are announced this week, the amount of salary in each case being given after the name of the institution :

Cheshire County Asylum, Macclesfield, Senior Assistant Medical Officer, unmarried, and not under 26 years of age—Salary £150 per annum, with board, &c.

Clayton Hospital and Wakefield Dispensary. Wakefield, Junior House-Surgeon, unmarried—Honorarium £25 per annum, with board, &c.

Paddington Green Children's Hospital, W., House-Surgeon. Appointment for six months—Salary at the rate of £55 5s. per annum, with board, &c.

Salop Infirmary, Shrewsbury, Dispenser—Salary £100 per annum, without residence or any extras.

University College Hospital, Resident Medical Officer.

### Non-Resident Medical Officers.

Borough of Leicester, Medical Officer of Health to act as Medical Superintendent of the Fever Hospital and Public Analyst—Salary £500 per annum.

Haverstock Hill and Malden Road Provident Dispensary, 132, Malden Road, N.W., Medical Officer.

London Society for Promoting Christianity amongst the Jews, fully qualified Medical Man as Medical Missionary, under 30 years of age—Salary £250 per annum, with furnished lodgings.

Seamen's Hospital Society, Greenwich, S.E., Visiting Physician for Branch Hospital in the Royal Victoria and Albert Docks.

Seamen's Hospital Society, Greenwich, S.E., Visiting Ophthalmic Surgeon.

Victoria Hospital for Children, Queen's Road, Chelsea, S.W., Anæsthetist, doubly qualified—Honorarium £50 per annum.

### THE STUDENT OF MEDICINE—(*continued*).

### PASS LISTS.

### University of Aberdeen.

GRADUATION IN MEDICINE, APRIL 4TH, 1892.

The following candidates have received degrees in Medicine and Surgery :—

DEGREE OF M.D.—C. G. Bennett, M.B., C.M., Buckie; E. Chambers, M.B., C.M., London; *A. R. Cushny, M.A., M.B., C.M., Fochabers; J. S. Dickie, M.B., C.M., Bournemouth; R. Eatough, M.B., C.M., Brindle; C. T. Ewart, M.B., C.M., London; *J. Galloway, M.A., M.B., C.M., London; A. C. Hutchings, M.B., C.M., Salisbury; W. Machain, M.B., C.M., Blackburn; A. D. Mackinnon, M.B., C.M., Skye; L. M. Scott, M.A., M.B., C.M., Aberdeen; †E. Stevenson, M.B., C.M., Birkenhead.

DEGREES OF M.B. AND C.M.—W. M. Anderson, Aberdeen; A. Baxter, Aberdeen; T. Brander, Garmouth; W. J. Carmichael, Aberdeen; A. B. Dalgetty, Auchinblae; N. B. Darabseth, Bombay; A. Davidson, Forres; W. R. Dugnid, M.A., Buckie; W. J. S. Ewan, Fyvie; D. Finlayson, Tain; A. Forbes, Leochel Cusnnie; A. Fraser, Inverness; T. H. Galbraith, London; A. G. Gall, Melbourne; A. Grant, Dufftown; J. W. Grant, Watten; E. M. Griffiths, Ruthin, North Wales; P. R. Ingram, Kildrummy; J. R. Keith, M.A., Bridge of Allan; R. E. Kerr, M.A., Bunchrew; A. Lamont, Ellon; J. Leach, M.A., Ardersier; D. D. Mackintosh, Aberdeen; W. Moir, Forgue; H. Mowat, Banff; J. Mowat, Auchnagatt; T. W. Ogilvie, Aberdeen; J. G. Parcoe, Littlehampton, Sussex; W. R. Pirie, M.A., Aberdeen; W. Ross, Fearn, Ross-shire; W. A. G. Russell, M.A., Orkney; J. Rust, M.A., Aberdeen; G. Savege, Montrose; C. R. Selbie, Pitcaple; R. R. Sutter, Timaru, New Zealand; A. W. Thompson, Jamaica; W. Trethowan, Victoria, Australia; H. L. De Vos, Ceylon; J. Wallace, M.A., Fearn, Ross-shire; A. Wilsden, Wooler, Northumberland; and R. Young, South Australia. G. K. Gifford, Shetland, has passed the examinations for the degrees of M.B. and C.M., but will not graduate until he attains the necessary age.

GRADUATION HONOURS.

The following gentlemen graduated with honours :—

HIGHEST HONOURS.—W. Trethowan.

HONOURABLE DISTINCTION.—A. Davidson, D. D. Mackintosh, T. W. Ogilvie, W. R. Pirie, and J. Rust.

The diploma in Public Health has been conferred on J. T. Wilson, M.B., C.M.Glasgow (with credit).

The following candidates have passed the First Division of the First Professional Examination for the Degrees of M.B. and C.M.: C. Bohrsmann, G. Brown, R. J. Brown, W. J. Byres, R. A. Coles, J. Dawson, J. Easton, J. S. Fraser, J. A. Gibb, J. Hadfield, J. Halley, P. J. Henderson, J. Laing, J. A. Mearns, ‡J. G. Milne, T. A. W. Ogg, W. M. Ogilvie, J. H. Patterson, A. C. Profeit, T. M. Ross, T. B. Trotter, J. M. Urquhart, and A. Wood.

The following candidates have completed the First Professional Examination: A. Archibald, E. Barnes, †J. A. Black, C. Bohrsmann, *T. I. Bonner, *G. Bruce, R. F. Campbell, A. L. Cobban, †R. Cruden, †A. Don, J. Easton, G. A. Gibb, J. A. Gordon, G. Grant, G. C. Grant, *W. C. Hossack, J. G. Jones, W. M. Keith, J. S. Laing, V. van Langenberg, ‡A. H. Lister, A. Low, *J. M. H. Macleod, J. MacPherson, J. S. Marr, P. Mitchell, *E. Oliphant, C. E. F. Owen-Snow, E. M. Payne, A. C. Profeit, A. Reid, G. Reid, *D. Ross, J. H. Rowe, A. P. Rust, †R. Smith, *G. Thomson, *W. Thomson, G. A. Troup, W. S. C. Waring.

The following candidates have passed the Second Professional Examination: F. S. Ainley, A. Alexander, *J. A. Allwood, W. Astin, R. Burgess, J. Cran, A. L. P. Cruickshank, W. Cruickshank, H. T. Dawson, W. R. Dugnid, D. G. Falconer, J. S. Findlay, H. Fraser, J. Fraser, F. A. Gill, J. H. Goodliffe, A. Gregor, W. Hector, J. Ingram, D. F. Justice, J. R. Kennedy, P. M. Lyon, R. G. MacGowan, G. Marr, C. R. Marrett, R. H. Marshall, T. Massie, †J. Matheson, *A. A. Moore, C. K. Morgan, P. M. Muttukumaru, A. Ogston, J. H. Patterson, E. Philip, J. F. Philip, A. Rajahsingham, M. W. Sharples, W. Shirreffs, A. M. R. Sinclair, A. Stables, W. Thomson, *G. J. A. Watson, T. D. Wingate, J. Wood, and T. M. Youngson.

The University Gold Medals have been awarded as follows: Fife Jamieson Memorial Gold Medal in Anatomy, A. W. Mackintosh, M.A.; Keith Gold Medal for Systematic and Clinical Surgery, P. Howie; Shepherd Memorial Gold Medal for Systematic and Practical Surgery, A. Stables; Matthews Duncan Gold Medal in Obstetrics, W. Trethowan.

* Theses worthy of "Highest honours." † Thesis worthy of "Commendation."

### University of Cambridge.

EXAMINATION IN SANITARY SCIENCE, APRIL, 1892.

The following candidates have satisfied the examiners in both parts of the examination: C. J. Bamber, M.R.C.S.London; T. H. Davison, M.B., C.M.Edin., London; R. H. Elliot, M.B., B.S.Lond., Kensington; W. Fligg, M.R.C.S., M.B.Edin., London; W. Foster, M.B.Camb., Shipley; W. J. Hadley, F.R.C.S., London; W. Hodges, M.R.C.S., Gloucester; J. T. Hyatt, M.R.C.S., Shepton Mallet; A. L. Jones, M.R.C.S., Swansea; J. H. Jones, L.S.A., Shipley; W. A. C. Roe, L.R.C.P.E., London; D. Semple, M.D., R.U.Ire., Netley; J. M. Taylor, M.R.C.S., L.R.C.P., Burslem; M. H. Taylor, L.R.C.P.E., L.R.C.S.E., Richmond; J. C. Thresh, M.B., B.C.Vict., Chelmsford; E. T. Trevor, M.R.C.S., London; G. W. Weir, M.D., M.A., R.U.Ire., South Shields.

* Indicates that the candidate has passed "with credit"; † indicates that the candidate has passed "with much credit"; ‡ indicates that the candidate has passed "with highest credit."

